# Evolution of Reproductive Division of Labor – Lessons Learned From the Social Amoeba *Dictyostelium discoideum* During Its Multicellular Development

**DOI:** 10.3389/fcell.2021.599525

**Published:** 2021-03-04

**Authors:** Ranjani Dhakshinamoorthy, Shashi P. Singh

**Affiliations:** ^1^Department of Biotechnology, Bhupat and Jyoti Mehta School of Biosciences, Indian Institute of Technology Madras, Chennai, India; ^2^Cell Migration and Chemotaxis Group, Cancer Research UK Beatson Institute, Glasgow, United Kingdom

**Keywords:** *Dictyostelium discoideum*, aggregation multicellularity, division multicellularity, ploidy, cell fate choices, reproductive division of labor

## Abstract

The origin of multicellular life from unicellular beings is an epochal step in the evolution of eukaryotes. There are several factors influencing cell fate choices during differentiation and morphogenesis of an organism. Genetic make-up of two cells that unite and fertilize is the key factor to signal the formation of various cell-types in due course of development. Although ploidy of the cell-types determines the genetics of an individual, the role of ploidy in cell fate decisions remains unclear. *Dictyostelium* serves as a versatile model to study the emergence of multicellular life from unicellular life forms. In this work, we investigate the role played by ploidy status of a cell on cell fate commitments during *Dictyostelium* development. To answer this question, we created *Dictyostelium* cells of different ploidy: haploid parents and derived isogenic diploids, allowing them to undergo development. The diploid strains used in this study were generated using parasexual genetics. The ploidy status of the haploids and diploids were confirmed by microscopy, flow cytometry, and karyotyping. Prior to reconstitution, we labeled the cells by two methods. First, intragenic expression of red fluorescent protein (RFP) and second, staining the amoebae with a vital, fluorescent dye carboxyfluorescein succinimidyl ester (CFSE). RFP labeled haploid cells allowed us to track the haploids in the chimeric aggregates, slugs, and fruiting bodies. The CFSE labeling method allowed us to track both the haploids and the diploids in the chimeric developmental structures. Our findings illustrate that the haploids demonstrate sturdy cell fate commitment starting from the aggregation stage. The haploids remain crowded at the aggregation centers of the haploid–diploid chimeric aggregates. At the slug stage haploids are predominantly occupying the slug posterior, and are visible in the spore population in the fruiting bodies. Our findings show that cell fate decisions during *D. discoideum* development are highly influenced by the ploidy status of a cell, adding a new aspect to already known factors Here, we report that ploidy status of a cell could also play a crucial role in regulating the cell fate commitments.

## Introduction

Across the evolution of eukaryotes, the occurrence of multicellular organisms remains the milestone (Koonin, 2010; Parfrey and Lahr, 2013; Williams et al., 2013; Umen, 2014; Brunet and King, 2017; Grochau-Wright et al., 2017; Fischer and Regenberg, 2019). The origins of multicellularity are explained by two basic cellular mechanisms. First: AM, as a stress response unicellular cells signal their near by cells and gather at one point to start functioning temporally as a single organized unit (Romeralo et al., 2012; Parfrey and Lahr, 2013). For instance, Myxobacteria, in situations of scarce nutrition, form swarms, mediated by intercellular signaling, which result in fruiting bodies (Sozinova et al., 2005; Mauriello et al., 2010; Lyons and Kolter, 2015). AM is a strategy also seen in the majority of the eukaryotic clades. This includes *Acrasis*, a division of Discoba (Brown et al., 2012a,b; He et al., 2014), *Fonticula alba*, a division of Holozoa (Brown et al., 2009), *Guttulinopsis* spp. in Rhizaria (Brown et al., 2012a,b), *Sorogena stoianovitchae*, a division of Alveolata (Lasek-Nesselquist and Katz, 2001; Sugimoto and Endoh, 2006), and *Copromyxa* and *Dictyostelia* spp., a division of Amoebozoa (Baldauf et al., 2000; Brown et al., 2011). The second mechanism is DM: Multicellularity is achieved by repeated division of a single cell. Sexual reproduction in fungi, plants, and animals is a good example for this strategy. In DM, multicellularity happens after a key step, where two haploid parents fuse to form a diploid zygote, which further undergoes several rounds of cell division to produce the complete organism.

Interestingly, these two strategies of multicellular evolution described here: AM and DM, are both part of the *Dictyostelium* life cycle (Weijer, 2004). *Dictyostelium* follows AM mode during developmental life and DM at sexual stage to enter multicellularity. During its asexual/developmental life cycle D. discoideum amoeba feed on microbes available in the soil and divide exponentially. Once starvation sets in, they signal and respond each other via cAMP signals and gather at a common point, undergo morphogenesis and differentiation to form a multicellular, terminally differentiated sorocarp (Raper, 1935; Eichinger et al., 2005; Schaap et al., 2006; Schaap, 2011; Du et al., 2015). *Dictyostelium* amoebae also comprise a sexual stage where two haploid cells of opposite mating type fuse to form a diploid zygote, which then attracts the haploid cells in the near surrounding. These haploid cells create the cellulose layer around the zygote before being cannibalized by the zygote, forming a complete macrocyst. After a period of dormancy, the macrocyst germinates releasing the haploid cells (Erdos et al., 1973; Okada et al., 1986; Bloomfield et al., 2010, 2019). This process is accompanied by haploid–diploid transition at appropriate intervals.

There are many intracellular cues being reported to influence cell fate decisions during *D. discoideum* development. Cells become either a stalk cell or a spore cell. The altruistic prestalk cells sacrifice their lives during the course of fruiting body construction, whereas the prespore cells become the dormant spores in the sori that later germinates to form the next generation of amoebae. The intracellular cues include cell-cycle phases (Weeks and Weijer, 1994; Azhar et al., 2001; Chen and Kuspa, 2005), intracellular Ca^2+^ levels (Azhar et al., 1995, 1996), nutritional status (Chattwood et al., 2013) of the cells at the time of starvation stress and the morphogens produced by the cells that take part in development, etc. (Schaap et al., 1995; Strmecki et al., 2005; Jang and Gomer, 2011). Our interest is to investigate whether ploidy status of the cells can also influence cell fate commitments during development, a cellular feature which, is elusive so far. In general, ploidy has a vast impact in nature, for instance, changes in ploidy levels bring changes in biomass production in plants (Tsukaya, 2008; Sun et al., 2011; Aversano et al., 2012; Cornellie et al., 2019), in honey bees. In several other hymenopterans’ ploidy serves as the sex determination factor (Heimpel and de Boer, 2008; Blackmon et al., 2017). The males/drones are haploids, whereas, the workers and the queens are diploids, further ploidy influences the reproductive division of labor.

Likewise, in higher metazoans, ploidy changes in the cells emphasize the division of metabolic/cellular and reproductive labors. The somatic cells are diploid and carry out regular cellular activities whereas, the germ cells are haploid and perform reproductive function, by which their genetic material is transferred to the next generation unlike the somatic cells. The dirty work hypothesis and several other reports build the concept that the metabolic functions carried out by the somatic cells lead to terrific mutations in their genetic material, which make them unsuitable as source of DNA to be passed on to the next generation. Therefore, the reproductive function is carried out by specially destined germ cells to generate mutation free healthy offspring (Gavrilets, 2010; Goldsby et al., 2014; Wahl and Murray, 2016). We are curious to learn whether such division of labor strategy does exist in *Dictyostelium*. The ploidy level could also here be a key regulator. As mentioned earlier, during development, *Dictyostelium* differentiates into two major cell types: the stalk cells and the spore cells. In comparison to somatic cells, the stalk cells sacrifice their lives for the benefit of spores, as germ cells like spores become the next generation amoebae. This appears comparable with the germ-soma distinction that we observe in many organisms. We believe, this study may help to gain new perspectives on the evolution of germ-soma distinction from the unicellular eukaryote to highly organized multicellular organisms.

We tested our hypothesis by reconstituting one of the haploid parents (wild type) with its isogenic diploid derivative and visualized their fates in the chimeric development structures, mainly at the migrating slug stage. Thus far, the natural isolates of *Dictyostelium* from various locations of the world are haploids and there are no reports on the existence of diploids in nature (Erdos et al., 1973). This could be due to lack of selection tools or appropriate markers to isolate the diploids in nature. However, the genome analysis studies spur that the occurrence of diploids in nature could be a frequent event (Flowers et al., 2010). Even though *Dictyostelium* achieves diploid state during its sexual stage, the zygote formation followed by macrocyst generation is a tedious and difficult to replicate state under laboratory conditions. Therefore, we exploited parasexual genetics, which is peculiar in fungi and single-celled organisms, to create *Dictyostelium* diploids (Williams et al., 1974; King and Insall, 2003). Under axenic laboratory conditions, the chances of diploid occurrence is one in thirty thousand cells (King and Insall, 2003). However, by applying appropriate selection pressures it is possible to fuse two different *Dictyostelium* strains. Unlike the sexual stage, in parasexual genetics the parent strains belong to same mating type and share similar genetic background except for the markers they carry. We generated the *Dictyostelium* diploids by following the parasexual genetics described in King and Insall (2003).

In the chimeric (haploid–diploid) developmental structures we observed that the haploids preferentially contributed to the spore population, which forms the next generation amoebae, dominating the diploids (Dhakshinamoorthy et al., 2017). This seems comparable to cell fate commitments in higher metazoans, where the zygote undergoes repeated cell division resulting in haploid germ cells whose DNA is passed on to the next generation, but not the genetic material of the diploid somatic cells. Our findings demonstrate that apart from several other reported factors, ploidy status of a cell serves as a key factor that influences the cell fate during *Dictyostelium* development.

## Materials and Methods

### *Dictyostelium* Haploid and Diploid Cultures

In this study, haploid and diploid strains of Ax2 and Ax3 background were used. The haploid parents Ax2 and IR150 (Ax2 background) were obtained from Robert H. Insall (Cancer Research UK Beatson Institute, Glasgow, United Kingdom) and their isogenic diploid strain, Ax2IR150 was generated following parasexual genetics in our laboratory. Further, the haploid parents Ax3, IR110 (Ax3 background), as well as their isogenic diploid derivative, Ax3IR110 were kind gifts from Robert H. Insall. Ax2 and Ax3 cultures were grown in HL5 medium. IR150 and IR110 are thymidine auxotrophs, achieved by inserting G418 resistance gene at the thymidine locus (*thy*A) of Ax2 and Ax3 strains, respectively. Therefore, IR150 and IR110 (henceforth mentioned as IR150^thy^ and IR110^thy^) need thymidine supplement for their growth in HL5 medium and are resistant to G418. These thymidine auxotrophs were grown in HL5 medium fortified with thymidine (100 μg/ml) and G418 (10 μg/ml). Ax2IR150 and Ax3IR110 diploids were grown in HL5 media containing G418 (10 μg/ml).

### *Dictyostelium* Transfection

Employing electroporation technique Ax2 and Ax3 haploids were transfected with the expression vector pTXRFP. 2 × 10^7^ cells were sedimented and washed twice with electroporation (EP) buffer. Later, cell pellets were resuspended in 100 μl EP++ buffer containing pTXRFP plasmid. The cell-DNA suspensions were transferred to pre-chilled electroporation cuvettes (5 mm gap) and stored on ice. The cells were pulsed at 300 V, 2 ms × 5 cycles of square pulses in 5 s duration using the BTX ECM 830 electroporator. Finally, cell suspensions were transferred to Petri dishes containing HL5 medium. After 24 h, G418 (10 μg/ml) selection pressure was added. EP buffer: 10 mM K_2_HPO_4_, 10 mM KH_2_PO_4_, and 50 mM sucrose, pH 6.2. EP++ buffer: 10 mM K_2_HPO_4_, 10 mM KH_2_PO_4_, 50 mM sucrose, 1 mM MgSO_4_, 1 mM NaHCO_3_, 1 mM ATP, 1 μM CaCl_2_, pH 6.2.

### Generation of Diploid Strain

**FIGURE 1:**
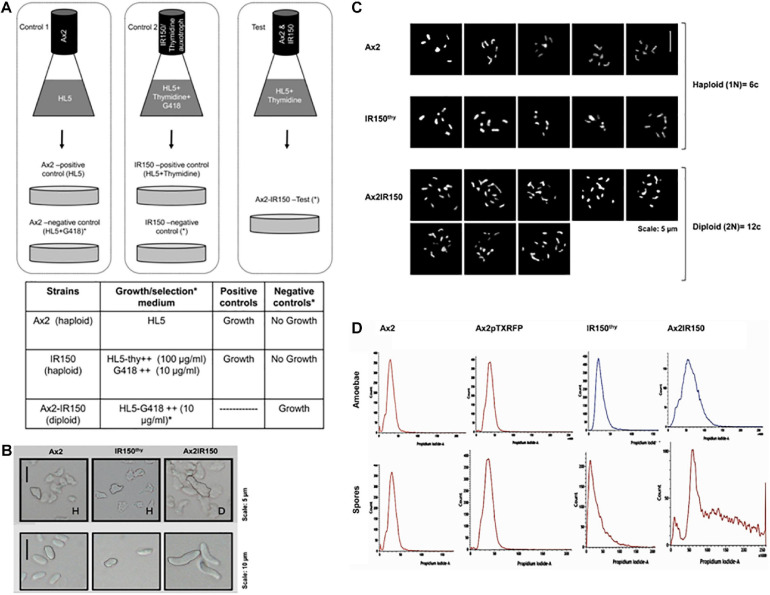
Confirmation of Ax2IR150 diploid. **(A)** Schematic representation of diploid generation: Pure parent strains are grown in control flasks as indicated and in test flask both the parents, Ax2 and IR150^thy^ mixed in equal ratio and grown in medium that supports their growth. After overnight incubation at 22°C/140 rpm, cultures in control flasks shifted to Petri dishes labeled positive and negative controls and test flask culture shifted to test plate. Positive control plates contain media that supports parent strains growth, media in negative control and test plates allow only diploid formation. Growth medium used and the expected outcomes are summarized in the table below. **(B)** Morphology of haploids and diploids: Axenically grown haploid and diploid amoebae allowed to adhere to Petri dishes and imaged under upright microscope (top panels), scale = 5 μm. Haploid and diploid amoebae subjected to development. After 48 h, spores harvested from haploid and diploid fruiting bodies and visualized under upright microscope (bottom panels), scale = 10 μm. **(C)** Karyotyping: Haploid parents and their isogenic diploid amoebae stained with giemsa and imaged under high resolution microscope, five images for each haploid strain and eight comparative images for diploid strain are displayed, scale = 5 μm. **(D)** Flow cytometry analysis of haploid parents and their isogenic diploid: Vegetative amoebae (top panels) and spores (bottom panels) were fixed with ice-cold methanol before flow cytometry analyses. The panels displayed indicate DNA content in the haploid and diploid strains. Ax2pTXRFP is the wild type Ax2 strain transfected with RFP reporter and used for reconstitution experiments.

The isogenic diploid between Ax2 and IR150^thy^ was generated by parasexual genetics as described in King and Insall (2003). Both haploid parents, Ax2 and IR150^thy^ were taken in equal ratios; 5 × 10^6^ cells each and mixed well before introducing into a flask containing 10 ml of HL5 medium supplemented with thymidine. The parent strains were also grown separately in their appropriate growth medium as controls, i.e., Ax2 resuspended in HL5 medium, IR150^thy^ in HL5 medium supplemented with thymidine. Cultures were incubated at 22°C 140 rpm for 12 h (Figure 1A). From the Ax2 and IR150^thy^ control flasks 0.5 ml aliquots of culture were removed and introduced into Petri dishes labeled as positive controls. Remaining cultures (9.5 ml) were transferred to two Petri dishes named as negative controls. The entire culture of the Ax2: IR150^thy^ mixed culture was transferred to a Petri dish labeled as test. The Petri dish cultures were left undisturbed for 1 h at 22°C to allow cells to adhere. Later, the existing medium in the positive control plates were replaced with fresh medium same as their respective flask cultures (HL5 for Ax2 and HL5+thymidine for IR150^thy^), whereas the negative controls and the test plates were replaced with full selection medium (HL5+G418), which allows only the diploids to grow.

### FACS Analysis

Flow cytometry (BD flow verse) analysis to distinguish the haploid and diploid strains were performed following the method described by King and Insall (2003). Haploid and diploid strains were grown in 90 mm (Tarsons) Petri dishes and harvested at 80% confluence. Cultures were sedimented at 500 × *g* for 7 min at 22°C, pellets were resuspended in 10 ml ice-cold methanol (−15°C) and stored at 4°C after vigorous vortexing for 30 s at RT. Spore masses harvested from 48 h old fruiting bodies were also analyzed, as elaborated above, but the spores were sedimented at 10,000 × *g* for 5 min at 22°C. On the day of flow analysis amoebae/spores were centrifuged and resuspended in 1 ml KK2 buffer (2.2 g KH_2_PO_4_ and 0.7 g K_2_HPO_4_ per liter). RNase A treatment was performed by adding 10 μl of 100 μg/ml RNase A and incubation at 22°C for 20 min. Subsequently, cells were stained with 10 μl of 100 μg/ml propidium iodide (PI) before subjecting to flow cytometry examination. Fixed samples were analyzed at a wavelength of 488 nm and minimum 10,000 events per sample were recorded. The fixed samples were stored no longer than 4 days at 4°C before flow analyses.

### Karyotyping

To visualize the ploidy status of the haploid and diploid amoebae, cytological staining was performed as described in King and Insall (2003). 5 × 10^6^ cells were seeded in a Petri dish (5 cm) loaded with acid washed coverslips and incubated at 22°C for 2 h with required supplements. Later, the Petri dishes were replaced with HL5 medium containing 33 μM Nocodazole and incubated at 22°C for 2 h. After incubation, coverslips were transferred to Petri dishes containing pre-chilled distilled water for 10 min. Cells were fixed with cold ethanol:glacial acetic acid (3:1; v/v) suspension for 1 h. The fixing step was repeated in fresh fixing suspension for 10 min. Finally, coverslips were air dried, mounted with DAPI containing mounting solution, and then visualized with a fluorescence microscope (Olympus BX51) at 100×/1.3 magnification.

### Microscopy

Single cells (amoebae): Haploid and diploid amoebae were cultured in 90 mm Petri dishes. When the monolayer had reached 90% confluence, the growth medium in the Petri dishes were discarded and the haploid and diploid amoebae were imaged using a 20× objective under the upright microscope (Nikon Eclipse 80i, CFI 10X/22). Spores: Sori of 48 h old haploid and diploid strains were gently touched with 200 μl microtips and resuspended in KK2 buffer. Spore suspensions were briefly mixed and mounted on glass slides before imaging under the upright microscope (Nikon Eclipse 80i CFI 10X/22). Slugs: Migrating slugs were imaged under the Stereo zoom microscope (Nikon SMZ1000, C-W10xB/22) and the upright microscope (Nikon Eclipse 80i, CFI 10X/22) at 16 h of development. Fruiting bodies: 24 h old fruiting bodies were allowed to gently fall on the agar surface without any manual disturbance before being imaged. Those fruiting bodies that were standing erect were also imaged under the Stereo zoom microscope (Nikon SMZ1000, C-W10xB/22).

### Carboxy Fluorescein Succinimidyl Ester (CFSE) Staining

Haploids and diploids were tracked during development using two different markers. The haploids were transfected with an expression vector that encodes the protein RFP. Attempts to transfect diploids were unsuccessful. Therefore, the diploids were tracked by staining them with the vital, fluorescent dye carboxy fluorescein succinimidyl ester (CFSE). We also performed the haploid–diploid reconstitution experiments by marking only one population (haploid or diploid) to confirm there are no artifacts caused by the markers used in the study. Amoebae were harvested from 90 mm Petri dishes (HiMedia) and sedimented at 500 × *g* for 7 min at 22°C. After washing with KK2 buffer, cell pellets were resuspended at a cell density of 1 × 10^7^ cells/ml. CFSE (Thermo Fisher Scientific) was added at a final concentration of 10 μM in a 2 ml micro centrifuge tube. The cell suspensions were incubated at 140 rpm for 1 h at 22°C in the dark. CFSE stock solutions (5 mM) were prepared in DMSO (Thermo Fisher Scientific) and stored at −20°C as described by the manufacturer.

### Reconstitution Experiments

Haploid and diploid amoebae grown in 90 mm Petri dishes were harvested by gently taping the plates. Cells were enumerated under the microscope to determine the cell densities. Haploid and diploid cell suspensions were prepared at a cell density of 1 × 10^7^ cells/ml in KK2 buffer. Haploid and diploid cells mixed at different ratios (1:1, 2:8, 1:9, 8:2, and 9:1) were deposited on KK2 agar (1%) plates (35 mm, Himedia) and incubated at 22°C in a dark, moist chamber until the slugs and fruiting bodies were formed. Pure populations of haploid and diploid amoebae were also plated as technical controls. Self-mixing controls (haploid–haploid and diploid–diploid) were considered to test whether the markers used in the experiments are influencing the cell fate commitments. Migrating slugs were imaged both under the Stereo zoom (Nikon SMZ1000, C-W10xB/22) and upright microscopes (Nikon Eclipse 80i CFI 10X/22) and fruiting bodies were imaged only under the Stereo zoom microscope. Classical mixing experiments were conducted with haploid and diploid strains obtained from Ax2 and Ax3 backgrounds. See Supplementary Table 1 for details on haploid and diploid strains and their mixing ratios.

### Glucose Assay

Glucose levels in haploid and diploid strains were quantified using a commercial kit following strictly the manufacturer instructions (GAHK20, Sigma-Aldrich). Amoebae grown under axenic condition were harvested at a cell density of 8 × 10^6^ cells/ml in KK2 buffer and incubated at 140 rpm, 22°C for 6 h. After incubation, amoebae were sedimented at 500 × *g*, 22°C for 7 min and stored at −80°C for 8 h. The samples were thawed to RT, 10 μl of supernatant was mixed with 90 μl of the glucose assay reagent, and incubated at RT for 15 min. Samples were subjected to spectrometer analyses at 340 nm absorbance, which is the direct measure of glucose content in the samples. Subsequently, 10 μl sample was used to measure the protein content in the sample using the Bradford reagent (BioRad). Finally, glucose content in the samples was quantified by normalizing with the total protein content in the samples.

## Results

### Diploids Generated by Parasexual Genetics

We generated the isogenic diploid strain Ax2IR150 between haploid parents Ax2 and IR150^thy^ by employing parasexual genetics. The parent strains Ax2 and IR150^thy^ cannot grow in the full selection medium (i.e., HL5+G418) and only the fused Ax2: IR150^thy^ diploid clones survive. While no signs of growth were observed in the negative control plates, the positive control plates reached confluence after 2 days of starting the plate. We isolated the clones that survived the full selection pressure and examined further to confirm their morphology and ploidy status under the microscope. As reported by other workers before, diploid amoebae are almost double in size compared to their haploid parents, when imaged under the microscope (Figure 1B). Similarly, the spores harvested from Ax2IR150 are double the size of their haploid parents (Figure 1B). Karyotyping analysis of the two haploid parents Ax2 and IR150^thy^ and their isogenic diploid Ax2IR150 displayed six and twelve chromosomes, respectively (Figure 1C). Further supporting the karyotyping results, when analyzed by flow cytometry, the diploid amoebae represented DNA peak intensities about twice that of their haploid parents as described by King and Insall (2003). We also examined the ploidy status of Ax2IR150, Ax2, and IR150^thy^ spores. The DNA peak intensities are similar to those obtained for haploid and diploid amoebae: The DNA of Ax2IR150 spores show DNA peak intensities almost double than from the haploid parent spores (Figure 1D). After confirming the ploidy status of Ax2, IR150^thy^ haploids and Ax2IR150 diploid offspring by different methods, these strains were used for the reconstitution experiments. Initially, we compared the developmental time frames of Ax2 and Ax2IR150 by allowing them to undergo the entire developmental cycle. The time points at which Ax2IR150 formed aggregates (8 h), mounds (12 h), slugs (16 h), and fruiting bodies (24 h) seems comparable to its wild type parent Ax2 (data not shown). Apart from the Ax2 background strains, we also used haploid and diploid strains of Ax3 background for our reconstitution experiments after scrutinizing them under the microscope and flow cytometry.

### Diploids Are Heavier Than Haploids

**FIGURE 2:**
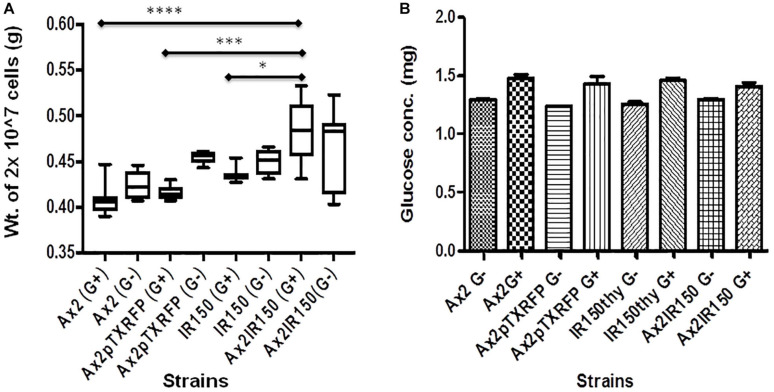
Intracellular characteristics of haploids and diploids. **(A)** Heaviness of haploid and diploid amoebae: Haploid and diploid amoebae grown in axenic medium fortified with glucose and without glucose, 10 ml culture of each strain was centrifuged at 2 × 10^6^ cells/ml density and washed with KK2 buffer. Pellets dried for about 20 min at 60°C and their dry weights measured and represented in grams (g). One-way ANOVA, Tukey’s multiple comparison test was performed to ascertain statistical significance, ^∗^*p* < 0.05, ^∗∗∗^*p* < 0.001, ^****^p < 0.0001, and *n* = 5. **(B)** Glucose quantification: Vegetative amoebae were grown as described earlier, followed by cell lyses and glucose quantification as suggested by the manufacturer. Glucose concentration in each strain is derived after normalizing with the total protein concentration.

To assess whether the difference in cell sizes also reflect in differences in their heaviness, we centrifuged equal number of Ax2, Ax2pTXRFP, IR150^thy^ and Ax2IR150 grown in axenic medium and weighed the dry pellets. The Ax2IR150 diploid grown in glucose fortified medium appeared heavier than its parents grown under similar conditions. The statistical significance for Ax2 and Ax2pTXRFP compared with Ax2IR150 represents ^****^p < 0.0001 and ^∗∗∗^*p* < 0.001 and for IR150^thy^^∗^*p* < 0.05, respectively. Similar trends were observed with the haploid and diploid strains grown in axenic medium lacking glucose, however, the differences are statistically not significant (Figure 2A). To understand whether the difference in heaviness between the haploid and diploid is due to differences in the intracellular metabolism, we quantified the intracellular glucose concentration. We found there is no significant difference in their intracellular glucose levels between the haploids and its isogenic diploid, irrespective in which growth medium the strains were grown (Figure 2B). Noticeably, IR150^thy^ remains heavier than Ax2 irrespective of the growth conditions (Figure 2A) but there is no significant change observed at the glucose level (Figure 2B).

### IR150^thy^ Formed Aberrant Slugs and Fruiting Bodies

**FIGURE 3:**
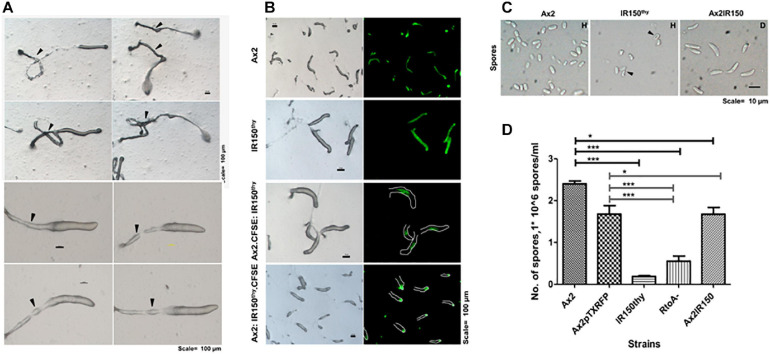
Aberrant developmental phenotypes of IR150^thy^. **(A)** Abnormal slugs and fruiting bodies of IR150^thy^: Arrowheads (black) depict migrating slugs of IR150^thy^ displaying thick and long slime sheaths (top, left panels), which later become fruiting bodies with extended stalks (top, right panels). High resolution images of IR150^thy^ migrating slugs (bottom panels). Arrowheads (black) indicate stout, extended slime sheaths, Scale = 100 μm. **(B)** Ax2: IR150^thy^ chimeric slugs: Ax2 and IR150^thy^ amoebae mixed at 1:1 ratio and deposited on KK2 agar plates for slug formation. Before mixing, either Ax2 or IR150^thy^ stained with CFSE. Appropriate controls, Ax2 and IR150^thy^ slugs stained with CFSE are also represented. Green color indicates location of CFSE stained population, Scale = 100 μm. **(C)** IR150^thy^ spore morphology: Spores obtained from Ax2, IR150^thy^, and Ax2IR150 fruiting bodies are depicted. Arrowheads (black) denote IR150^thy^ spores, **(D)** Spore formation efficiency (SFE) quantification: The number of spores obtained after plating 3.2 × 10^6^ amoebae for the strains Ax2, Ax2pTXRFP, IR150^thy^, RtoA-, and Ax2IR150 are represented. One-way ANOVA, Tukey’s multiple comparison tests were performed to determine statistical significance. ^∗^*p* < 0.05, ***p < 0.001, and *n* = 3.

When subjected to development, IR150^thy^ formed slugs at sixteenth hour, but upon migration, these slugs form stout and long slime sheaths. Overtime these slugs become small. Subsequently, the formed fruiting bodies demonstrated stick like extensions at the basal disk regions of the stalk as shown in Figure 3A. We reconstituted IR150^thy^ with Ax2 (1:1) and visualized the resulting slugs under the microscope. IR150^thy^ always preferred the prestalk pathway, however, the number of IR150^thy^ occupying the prestalk region is less than fifty percent of the slug (Figure 3B). In addition, the spores of IR150^thy^ are unhealthy (Figure 3C) and the spore formation efficiency (SFE) is drastically reduced in IR150^thy^ when compared with other haploids and diploids. When the SFE of IR150^thy^ is compared with Ax2, the statistical significance represents ^∗∗∗^*p* < 0.001 (Figure 3D).

### Haploids Gathered at the Aggregation Centers

**FIGURE 4:**
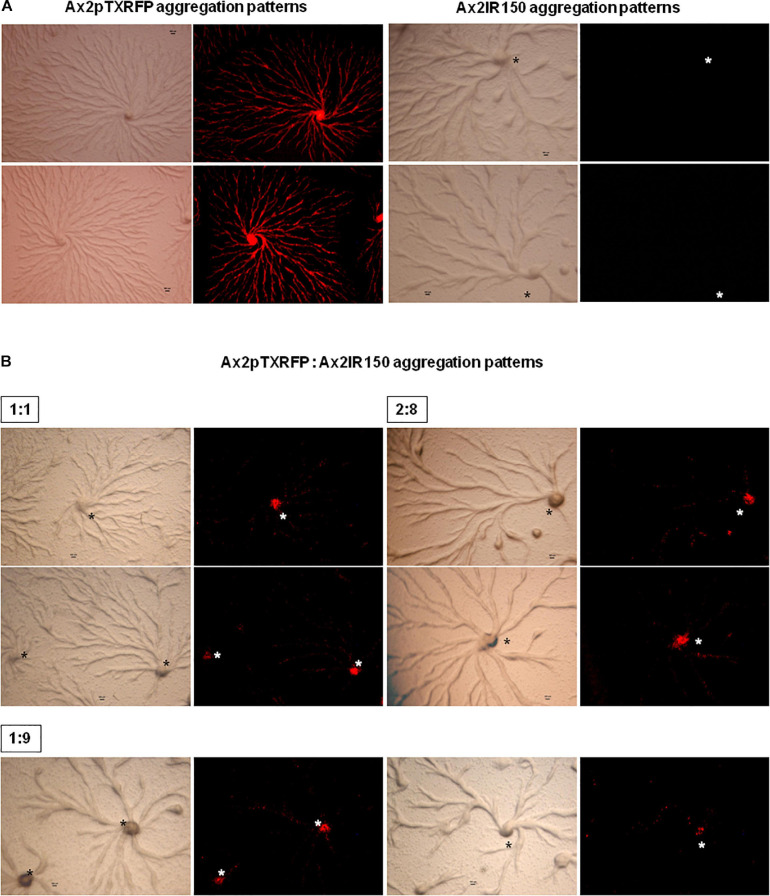
Ax2pTXRFP: Ax2IR150 aggregation patterns. Haploid parent, Ax2pTXRFP mixed with its isogenic diploid, Ax2IR150 in various ratios **(B)** 1:1, 2:8, and 1:9; deposited on KK2 agar plates and incubated at 22°C. After eighth hour of development, chimeric aggregates visualized under stereo zoom microscope. Aggregates of appropriate controls **(A)**, Ax2pTXRFP and Ax2IR150 are also displayed. Red color represents Ax2pTXRFP, asterisks (black and white) mark the aggregation centers, scale = 100 μm.

**FIGURE 5:**
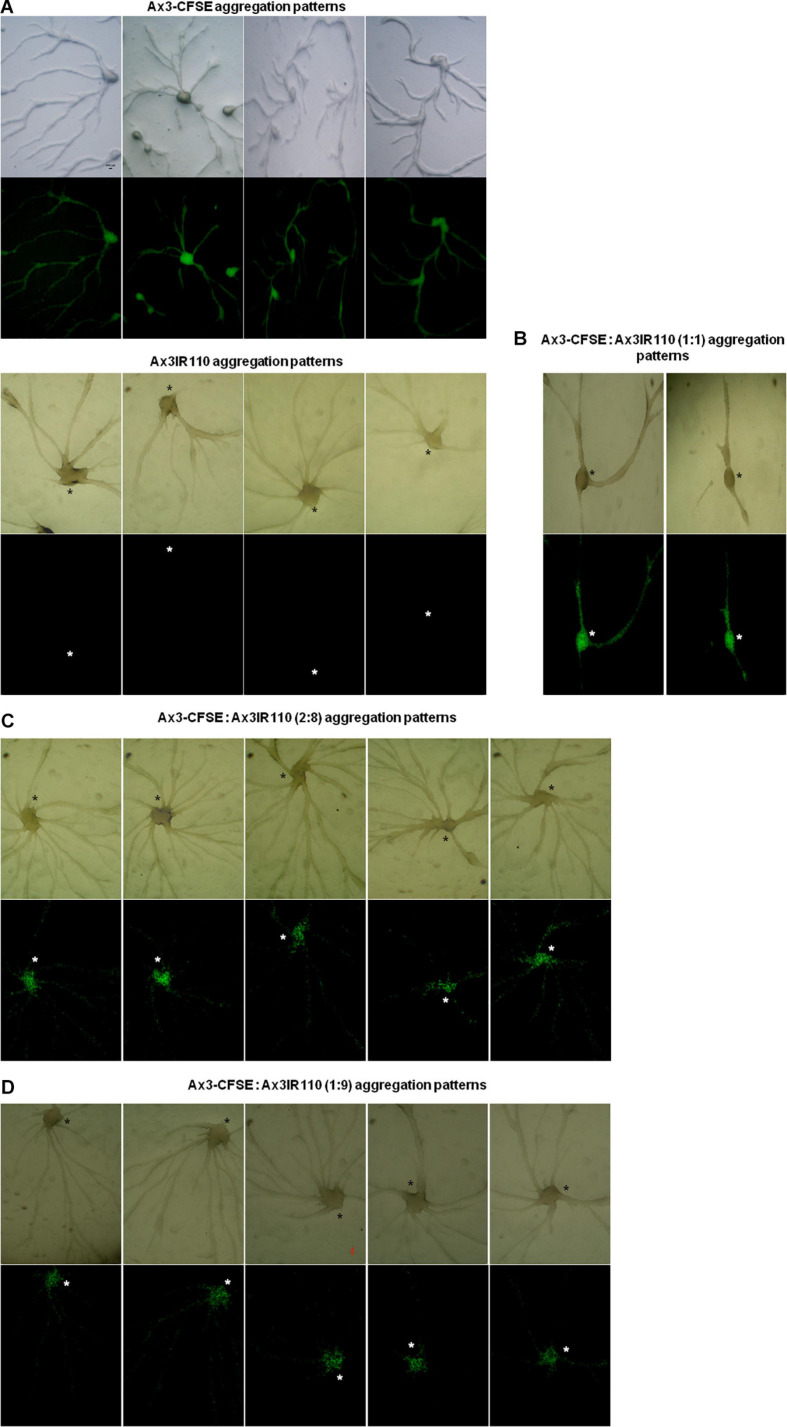
Ax3-CFSE: Ax3IR110 aggregation patterns. Ax3 amoebae stained with CFSE, washed and mixed with Ax3IR110 amoebae in different ratios 1:1 **(B)**, 2:8 **(C)**, and 1:9 **(D)**, deposited on KK2 agar for development, and imaged at aggregation stage. Appropriate controls **(A)**- Ax3-CFSE and Ax3IR110 aggregation patterns are also displayed. Green color represents Ax3-CFSE, asterisks (black and white) mark the aggregation centers, scale = 100 μm.

The haploid parent Ax2pTXRFP constituted with the diploid Ax2IR150 in various ratios (1:1, 2:8, and 1:9) and were allowed to enter the development cycle. At eighth hour of development, we visualized the chimeric aggregates under the stereo zoom microscope. Irrespectively of the mixing ratios, Ax2pTXRFP haploid preferentially localized at the aggregation centers and very scanty numbers are scattered in the cell streams. In contrast, Ax2IR150 is widespread in both: cell streams and aggregation centers (Figures 4A,B). To rule out the possibility that the intergenic marker being the cause for Ax2pTXRFP localization at the aggregation centers, we followed another approach: we stained the haploid population with the vital, fluorescent dye CFSE and reconstituted with the diploid strain Ax2IR150. The result obtained for Ax3-CFSE reconstituted with Ax3IR110 is summarized in Figures 5A–D. As observed in the Ax2pTXRFP: Ax2IR150 aggregation patterns, Ax3-CFSE remain localized at the aggregation centers, while Ax3IR110 remained scattered in both: cell streams and aggregation centers (Figures 5A–D).

### Haploids Prefer Prespore Regions in Haploid–Diploid Chimeric Slugs

**FIGURE 6:**
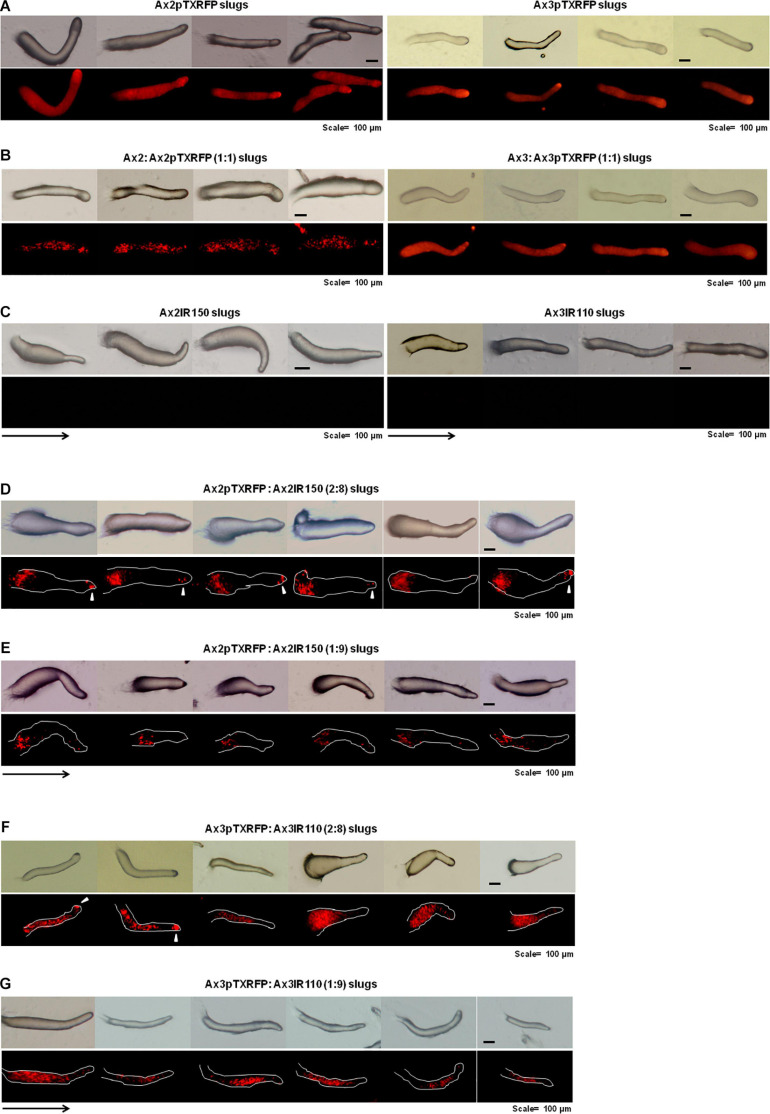
Chimeric slugs of RFP marked haploids: unmarked diploids. Axenically grown Ax2pTXRFP and/or Ax3pTXRFP haploid amoebae mixed with Ax2IR150 and/or Ax3IR110 diploid amoebae in different ratios (2:8 and 1:9) and imaged under stereo zoom microscope at slug stage **(D–G)**. Respective pure and self-mixing controls slugs are also displayed **(A–C)**, *n* ≥ 30 slugs. Red color indicates haploids; arrowheads (white) denote haploids at the signaling tip, and arrows (black) indicate slug directions, scale = 100 μm.

**FIGURE 7:**
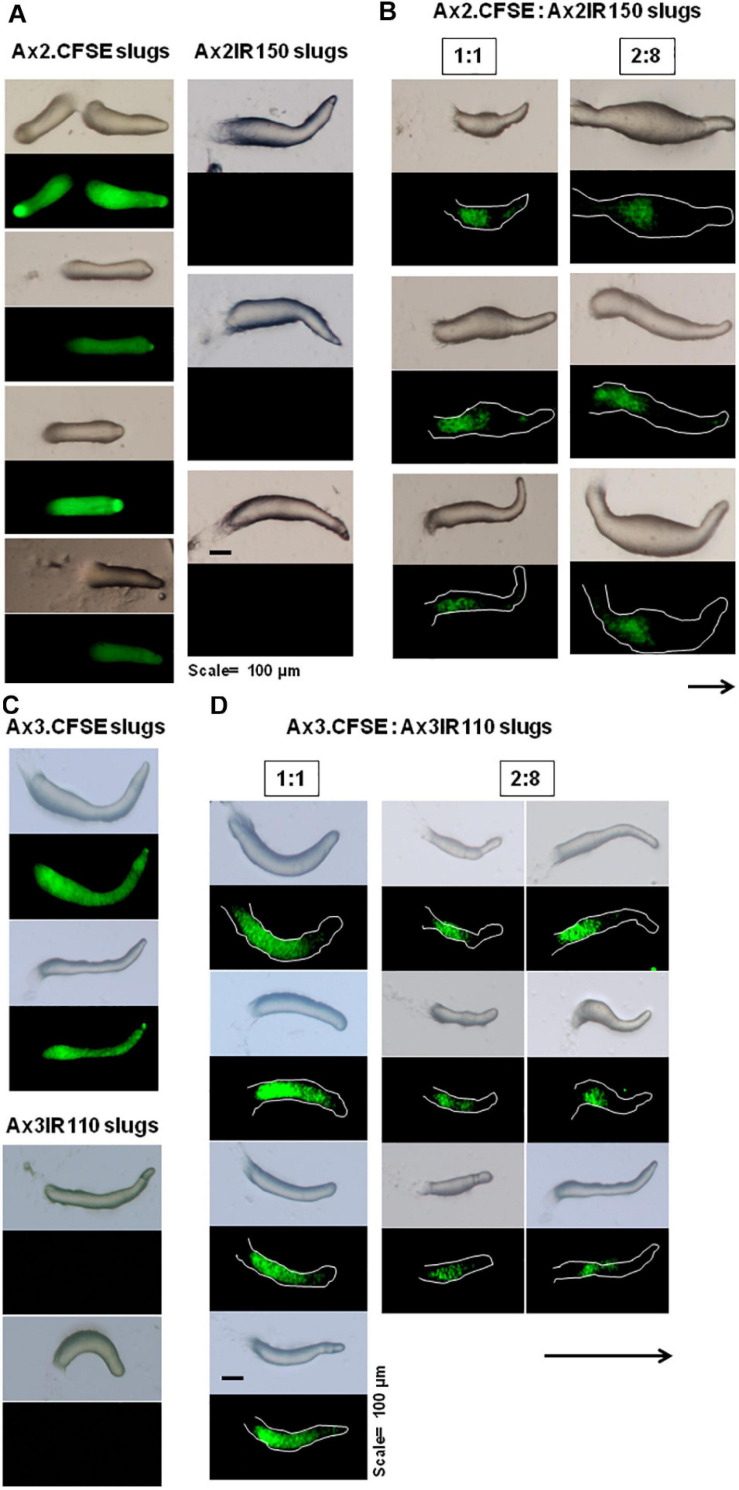
Chimeric slugs of CFSE marked haploids: unmarked diploid. Axenically grown Ax2-CFSE and/or Ax3-CFSE haploid amoebae mixed with Ax2IR150 and/or Ax3IR110 diploid amoebae in different ratios (2:8 and 1:9) and imaged under stereo zoom microscope at slug stage **(B,D)**. Respective control slugs are also displayed **(A,C)**. *n* ≥ 30 slugs. Arrows indicate slug direction, scale = 100 μm.

**FIGURE 8:**
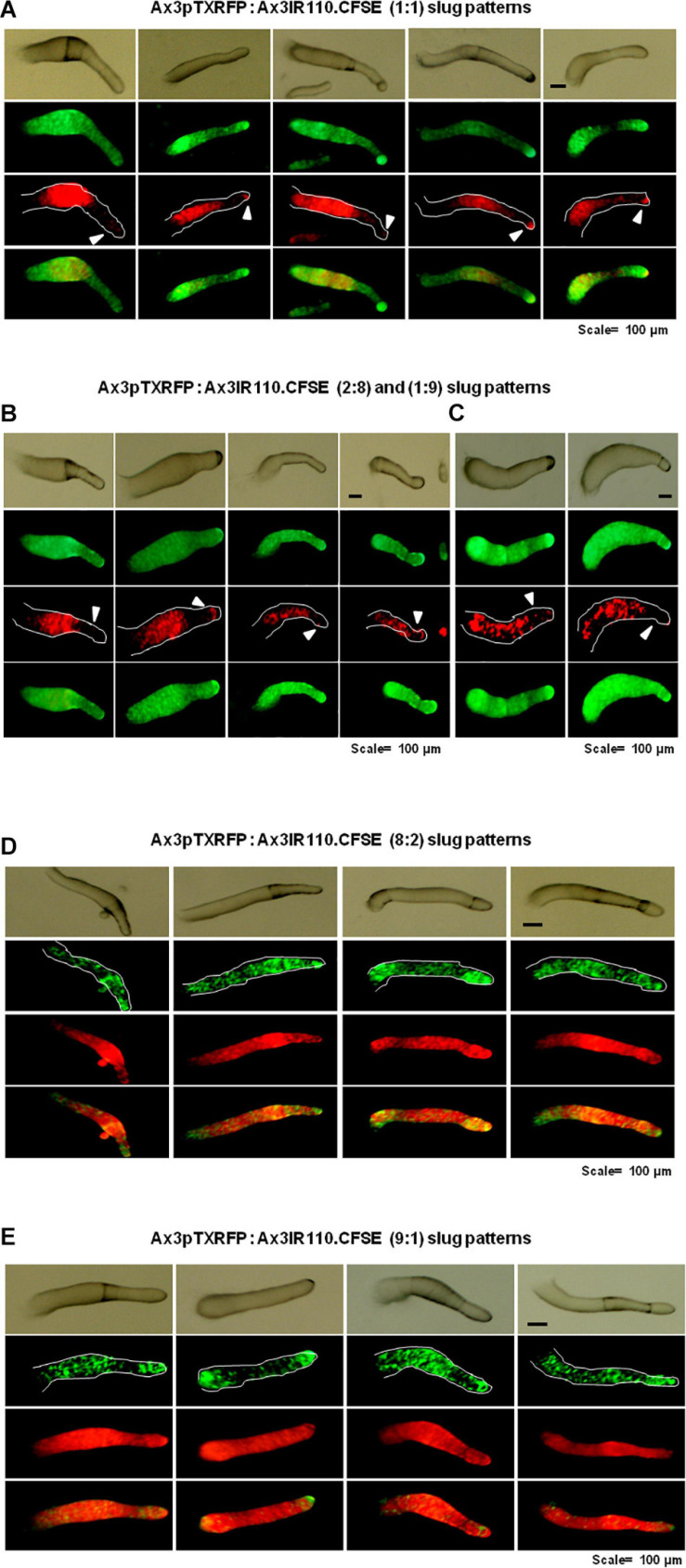
Chimeric slugs of Ax3pTXRFP: Ax3IR110.CFSE stained diploid. Axenically grown Ax3pTXRFP mixed with Ax3IR110.CFSE stained amoebae at different ratios 1:1 **(A)**, 2:8 **(B)**, 1:9 **(C)**, 8:2 **(D)**, and 9:1 **(E)** and imaged under stereo zoom microscope at slug stage. *n* ≥ 30 slugs. Red indicates the location of Ax3pTXRFP haploids, green represents the location of Ax3IR110.CFSE diploids, and orange color represents the merge locations of Ax3pTXRFP and Ax3IR110.CFSE. Arrowheads (white) indicate haploids at the signaling tip. scale = 100 μm.

When the wild type haploid parent Ax2pTXRFP and/or Ax3pTXRFP were mixed with their respective isogenic diploids Ax2IR150 and/or Ax3IR110, always the haploids tend to occupy the prespore regions in the chimeric slug structures. Irrespectively of the mixing ratios- 1:1, 2:8, and 1:9, haploids were predominantly found in the posterior slug regions and also a very minor portion of the signaling tip at the prestalk region representing a scanty distribution of haploids (Figures 6D–G). The slug patterns of Ax2pTXRFP and Ax3pTXRFP haploids and Ax2IR150 and Ax3IR110 diploids are also represented as 100% controls (Figures 6A,C). To confirm that the Ax2pTXRFP and/or Ax3pTXRFP haploid strains autonomously influence prespore fate, Ax2pTXRFP and/or Ax3pTXRFP strains are mixed with Ax2 and Ax3 strains, respectively, at a 1:1 ratio. These self-mixing controls imply a mosaic pattern of distribution of labeled (Ax2pTXRFP and/or Ax3pTXRFP) and unlabeled (Ax2 and/or Ax3) cells throughout the slug (Figure 6B). We also stained the haploids with CFSE before mixing them with unmarked diploids and subsequently allowed them to form slugs. As observed in haploids marked with RFP, independent of the mixing ratios, the CFSE marked haploids occupied the prespore slug regions. However, in this approach haploids occupying the prestalk signaling tip was a rare event (Figures 7A–D). Similarly, we reconstituted haploids carrying the RFP marker with CFSE stained diploids. We allowed the mixed cells to form slugs and imaged under the microscope. The double-labeled chimeric slug patterns remained comparable to the reports above. This approach clearly demonstrates that haploids predominantly occupy the prespore regions and the diploids are scattered across the prestalk and prespore slug regions (Figures 8A–D).

### Haploids Predominantly Occupied the Spore Mass in Fruiting Bodies

**FIGURE 9:**
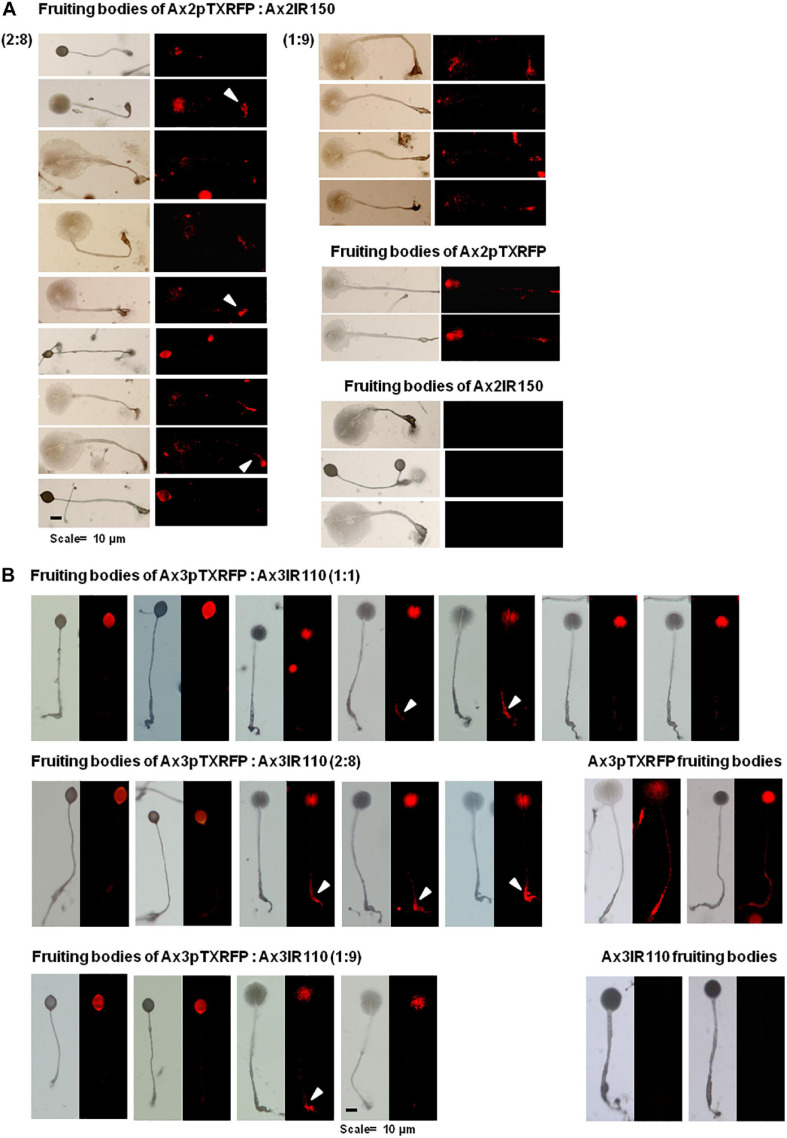
Chimeric fruiting bodies of Ax2pTXRFP: Ax2IR150. **(A)**. Haploid parent, Ax2pTXRFP amoebae mixed with its isogenic diploid, Ax2IR150 in different ratios- 1:1, 2:8, and 1:9) and deposited on KK2 agar plates. After 48 h incubation at 22°C, fruiting bodies imaged under stereo zoom microscope. Appropriate controls, Ax2pTXRFP and Ax2IR150 fruiting bodies are also displayed. Red represents Ax2pTXRFP haploid localization in the chimeric fruiting bodies. Arrowheads (white) indicate Ax2pTXRFP at basal disks of chimeric fruiting bodies. *n* ≥ 30 fruiting bodies, scale = 100 μm. **(B)** Ax3pTXRFP: Ax3IR110 chimeric fruiting bodies. Haploid parent, Ax3pTXRFP amoebae mixed with its isogenic diploid, Ax3IR110 in different ratios (1:1, 2:8, and 1:9) and allowed forming fruiting bodies as described above and chimeric fruiting bodies imaged under stereo zoom microscope. Appropriate controls, Ax3pTXRFP and Ax3IR110 fruiting bodies are also displayed. Red represents Ax3pTXRFP haploid localization in fruiting bodies. Arrowheads (white) indicate Ax3pTXRFP visualized at the basal disks of the chimeric fruiting bodies. *n* ≥ 30 fruiting bodies, scale = 100 μm.

**FIGURE 10:**
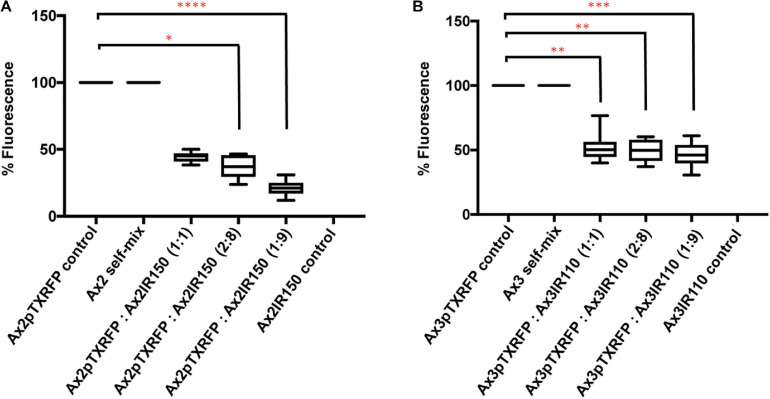
Fluorescence quantification in the slugs of Ax2 and Ax3 (haploid: diploid) strains. **(A,B)** Fluorescence quantification: Fluorescence across the slugs examined using ImageJ. Bars in the control (Ax2pTXRFP/Ax3pTXRFP) represent 100% fluorescence. Bars in 1:1, 2:8, and 1:9 represent percentage fluorescence observed at slug posterior. Around 15 slugs were quantified for each chimeric mix and for the controls. Dunn’s multiple comparisons test was performed to determine statistical significances. ^∗^*p* < 0.05, **p < 0.01 (1:1, 2:8), and ***p < 0.001.

When Ax2pTXRFP and/or Ax3pTXRFP were reconstituted with their corresponding isogenic diploids Ax2IR150 and/or Ax3IR110 and allowed forming chimeric fruiting bodies, the haploids preferentially contributed to the spore mass (Figures 9A,B). Additionally, a scanty population of haploids were visualized at the basal disk regions of the stalk. The images displayed include those fruiting bodies fallen while on the agar surface and undisturbed erected fruiting bodies. The fruiting bodies resulted from Ax2pTXRFP and Ax3pTXRFP haploids, and Ax2IR150 and Ax3IR110 diploids are represented as 100% controls (Figures 9A,B). Additionally, we also quantified fluorescence intensities at the slug posterior using imageJ (Figure 10), which also clearly demonstrated the haploid localization at slug posterior in both Ax2 and Ax3 slug chimeras. The statistical significance for Ax2pTXRFP: Ax2IR150 (1:9) represents ^****^p < 0.0001; and for Ax3pTXRFP: Ax3IR110 represents ^∗∗^*p* < 0.005 (1:1, 2:8) and ^∗∗∗^*p* < 0.001 (1:9), respectively.

## Discussion

The aim of our study was to understand whether ploidy status of a cell can influence cell fate commitment during *D. discoideum* development. We tested our hypothesis by reconstituting haploid and diploid amoebae at different ratios and allowing the cell mixture to form chimeric morphogenetic structures. We visualized the chimeric aggregates, slugs, and fruiting bodies at 8, 16, and 24 h, respectively. Our results clearly demonstrate that ploidy status of the cells strongly influences the cell fate commitment during *D. discoideum* development. The haploids showed clear preference for the spore pathway, starting from aggregation stage. The haploids are crowded at the aggregation centers and later at the slug posterior regions, which terminally differentiate into spores when reaching the fruiting body stage. For all our experiments, we included self-mixing controls to ensure that markers aren’t influencing the developmental cell fates. We think, the chimeric morphogenetic structures obtained by mixing the haploid–diploid amoebae appear comparable to the germ-soma distinction that we see in higher metazoans. The germ cells are haploid whose DNA gets transferred to next generation (reproductive function) unlike the somatic cells that are diploid, which sacrifices itself for metabolic functions.

We tracked the cell fate of haploid amoebae that seems comparable with the germ cells and the diploids that mimics the somatic cells at various morphogenetic stages of *D. discoideum* development. This way we obtained the distinct localization of haploids and diploids in the chimeric aggregates, slugs and fruiting bodies. As demonstrated, during development of pure haploid populations, as well as haploid:diploid chimeras, developmental cell fate commitment arose starting at the aggregation stage. Independent of the labeling method adopted or the mixing ratios, the haploids were found clustered at the aggregation centers. Earlier work by Huang et al. (1997) demonstrates that only those cells, which initiate development, localized to the aggregation centers and eventually became spores. Additionally, they differed in their cell-cycle phase at the time of starvation and in their developmental gene expression patterns when compared with prestalk cells. In this study, Huang et al. (1997) had mixed the wild type Ax2 cells with a strain NP160, which is defective to initiate developmental signaling, but is capable of sensing the developmental signals that other cells generate. Another study conducted by Araki et al. (1994) also obtained similar results. They have mixed the synchronized population of cells that have the tendency to become prespore cells with non-synchronized population and found that cells at the aggregation centers had the propensity to become the prespore cells in the slugs. These two studies remain comparable to our results, in which the haploids of the chimeric haploid:diploid mix localized to the aggregation centers. However, later, at the slugs stage the haploids become isolated to the slug posterior, ranging from mid to rear most slug posterior regions. This appears different from the developmental pattern of pure haploid population, where the prestalk:prespore ratios in the slugs are roughly maintained at 20:80%. In other words, one-fourth of the slug front is occupied by the prestalk cells and the posterior three-fourth of slug rear is occupied by the prespore cells. At the current stage we can’t explain this change in prestalk:prespore ratio. Noticeably, we observed that the relative spore formation efficiency of haploids was higher than the pure diploid population (Figure 3D).

Even though in the haploid–diploid chimeric slugs the haploids are mostly restricted to the slug posterior, a scanty distribution of haploids is visualized at the prestalk regions. In particular, at the signaling tip region where the pstA and pstAB cells are usually localized. Supporting this observation, subsequently, these scanty populations localized to the inner and outer regions of the basal disk in the mature fruiting body. It is also possible that these scanty populations are anterior-like cells (ALCs), which initially sorted to the prespore region, got reprogrammed and were relocated to the prestalk regions during morphogenetic cell movement. The ALCs initially form the upper cup and lower cup cells, which cradle the spore mass, but terminally differentiate into cells at the outer regions of the basal disk (Williams, 2006).

In our study, we have used cells of two different sizes. The diploid cells are approximately double the size of haploids. Hence, cell size might also influence cell-fate. There are many reports in the past about the role of cell-size on cell-fate. The research on cell-size influence over cell-fate conducted by Bonner and Frascella (1953), and Bonner (1959) had raised evidences that the anterior prestalk cells are larger than posterior prespore cells based on cell sizes measurements at the anterior and posterior slug regions. Following this, Takeuchi’s study (1969) had also proved that preaggregation differences exist among the cells. When the preaggregation cells were centrifuged two cell patches resulted based on density. The heavier (larger) cells occupied the slug anterior or formed the prestalk cells. Whereas the lighter (smaller) cells occupied the slug posterior or formed the prespore cells. Bonner’s study (1971) reproduced the same results. Two approaches were carried out in this work. (1) A part of slug anterior and a part of slug posterior were removed and allowed to form fruiting bodies and their spores were measured. (2) Preaggregation cells were separated in dextran solution by centrifugation; heavy and light cells were separated and allowed to fruit. This study proved that spores derived from dense cells correspond to spores derived from anterior cells. However, this study brought to light that cell-size did not correlate with position of cells in the slugs always. This study was carried out using three strains namely, Dd-1, NC-4(S), and NC-4(L). The strains Dd-1 and NC-4(S) produce smaller spores when compared with NC-4(L) strain. The experiments with Dd-1 cells showed results supporting Bonner’s (1959) and Takeuchi’s (1969) findings; in the contrary, when NC-4(S) and NC-4(L) cells were mixed the results were opposite. The smaller (less dense) cells occupied the prestalk region and the larger (heavy cells) occupied the prespore slug regions. Hence, the study conducted by Bonner et al. (1971) concluded that cell size cannot be a universal cell-fate determination factor during *D. discoideum* development. Supporting this finding, Maeda and Maeda (1974) reported that preaggregation cells that were separated by isopycnic centrifugation represented two distinct cell fractions, the light and heavy cells. The light cells localized to the anterior prestalk region and the heavy cells formed the prespore part of the slug.

Similarly, Schaap et al. (1982) described a different characteristic of the heavy and light cells, the electron density. This study proved that the anterior presalk cells are electron dense and are small, whereas the posterior prespore cells represented inter-mixed electron densities. The studies on cell heterogeneity conducted by Ratner and Borth (1983) following density centrifugation revealed that majority of the cells in the slug posterior demonstrated expected prespore characteristics but there were also lighter (minor) density cells resembling anterior prestalk cells.

These studies describe totally contradicting results to earlier reports by Bonner and Takeuchi. Considering all these studies, we conclude that cell size may not be a key factor that could influence cell-fate during *Dictyostelium* development. Moreover, in our study, none of our reconstitution experiments ended up in stalky or spory fruiting bodies irrespective of the fact that diploids are heavier (larger) than the haploids and the various haploid:diploid mixing ratios (1:1, 2:8, 1:9, 8:2, and 9:1).

Also, the density-based cells separation on performed, linear Percoll density gradient represented vegetative cells belong to different cell densities and they vary in their DNA content as well (Weijer et al., 1984c). Additionally, this study raised a notion that there could be some cell-cycle linked differences among the vegetative cells, which could bias their commitment toward prestalk or prespore fate.

Concerning the heaviness property of prestalk and prespore cells, during *Dictyostelium* developmental, the prespore cells show higher protein levels compared to prestalk cells and different levels of enzymes, such as UPD galactose-polysaccharide transferase, cAMP phosphodiesterase, glycogen phosphorylase, etc. (Tsang and Bradbury, 1981). These properties influence the spatial-temporal differences in the prespore and prestalk cells. In contrast, when the heaviness property of haploid and diploid cells is compared, the diploids are heavier than the haploids, but are scattered across the entire stretch of the slugs (Figure 8E), whereas the haploids remain restricted to the posterior slug regions. This observation suggests that there are additional key factors influencing cell fate.

There are several reports that the nutritional status of a cell could play a crucial role in cell fate commitments at *Dictyostelium* development. Concisely, several workers have proven that when cells grown at two different nutritional conditions, are reconstituted and finally allowed to enter the developmental cycle, there appears a clear distinction in the spatial distribution of these cells in the morphogenetic structures. For instance, when cells grown in glucose fortified medium were mixed with those cells grown in the medium lacking glucose and allowed forming fruiting bodies; chiefly, those cells grown in glucose fortified medium occupied the spore mass, whereas the stalk was made from those cells grown in medium lacking glucose (Leach et al., 1973; Thompson and Kay, 2000; Sistla et al., 2012). Based on this observation, we speculated a difference in glucose reserves between the haploids and diploids, as the diploids are double the size of haploids. However, we couldn’t find any significant difference in the intracellular glucose levels between the haploids and diploids, irrespective of the growth conditions. Anyhow, the diploids were heavier than the haploids, which correlate with their difference in cell morphology. Apart from the morphological and physiological difference observed between the haploids and diploids, the core difference is at the DNA level. The diploids constitute double the number of chromosomes than the haploids (as confirmed by the flow cytometry and karyotyping) however; the haploids predominantly occupy the prespore slug regions and become the spores in the fruiting bodies. Considering the diploids as the next generation amoebae might pave way for the accumulation of extra nuclear DNA; therefore, might add up to a burden of the molecular machinery of the cell. Hence, the haploid amoebae are chosen for the spore pathway, which becomes the next generation amoebae.

The reason why diploids are randomly scattered across the prestalk and prespore slug regions is yet to be answered. This observation implies that apart from the quantity of DNA, there could be some other factor, which is precisely controlling the cell fate choice during development. It is already reported by many researchers that the cell-cycle phase at the time of starvation could be the primary factor, which controls cell fate commitment beyond the other physiological factors (McDonald, 1984; Weijer et al., 1984a,b; Gomer and Firtel, 1987; Ohmori and Maeda, 1987; Zimmerman and Weijer, 1993; Araki et al., 1994; Gomer and Ammann, 1996; Azhar et al., 2001; Chen and Kuspa, 2005). In brief, the *Dictyostelium* amoebae grown under axenic condition display an 8 h cell-cycle with poorly detectable G1 phase as in some fungi (Weijer et al., 1984a,b). In *Dictyostelium*, G2 phase spans longer as the S phase and M phase lasts only for about 40 min (Zada-Hames and Ashworth, 1978). When synchronized, labeled population were mixed with unsynchronized, unlabelled cells the following cell fates were obtained. Cells at S and early G2 phase at the time of starvation localized to prestalk slug regions and those at the late G2 phase formed the prespore cells (Ohmori and Maeda, 1987; Araki et al., 1994; Maeda et al., 1997). It is also reported that when the cells at S or early G2 subjected to development, the slugs formed had abnormally higher percentage of prestalk cells (Weijer et al., 1984b; Wang et al., 1988). Interestingly, in many other organisms cell-cycle based regulatory mechanisms have been reported (Ambros, 1999; Jan and Jan, 2001; Remaud et al., 2008). Notch signaling plays an important role in cell fate determination in many organisms. For example, Notch signaling in *C. elegans* (during vulva formation) happens only during S phase (Ambros, 1999). At *Drosophila* bristle formation, cells are highly responsive to Notch signaling during S phase (Remaud et al., 2008).

We have used isogenic haploid and diploid strains that share similar genetic background in our study. Hence, we strongly believe that both haploid and diploid strains share common cell-cycle phases. If there are going to be any change in the cell-cycle it could be attributed to the increased ploidy in the diploids. Interestingly, the work on *rtoA-* is a proof to denote cell-cycle is not the only key factor that control cell-fate choices during *Dictyostelium* development. *RtoA-* has a typical cell-cycle like wild-type cells, but the cell-fate choices happen randomly at different phases of cell-cycle unlike the wild-type cells. And the studies by Wood et al. (1996); Chen and Kuspa (2005), and Jang and Gomer (2011) suggests that there may be parameters other than cell-cycle, which influences cell-fate in *Dictyostelium*. The recent work by Gruenheit et al. (2018) have demonstrated the changes in cell-cycle dynamics due to changes in nutritional conditions and its impact on cell-fate determination. As we grow both the haploids and diploids in same nutritional condition, we cannot take this into account for any dramatic changes in cell-cycle dynamics at the moment. Therefore, we believe that ploidy remains as the key factor that controls cell-fate during development in our haploid:diploid chimeric mixing experiments. However, we strongly believe that the live cell imaging studies carried out by Muramoto and Chubb (2008) to understand the cell cycle dynamics of the haploids during development might be very useful for us to understand the cell cycle dynamics in the diploids. We would conclude that studies on cell cycle regulation in diploids and further experiments adopting cell cycle synchronized haploid:diploid reconstitution may provide a clearer picture on the haploid:diploid pattern formation during chimeric development. Additionally, it could be informative to consider other physiological parameters, such as intracellular Ca^2+^ levels and cytosolic pH regulation in the diploids, which we will consider for our future studies. In haploids these two parameters are being regulated according to the cell cycle. Cells at S or early G2 phase at the time of starvation display higher Ca^2+^ levels and differentiate into prestalk cells. Whereas, the cells at mid-late G2 phase represent relatively low Ca^2+^ levels and differentiate into prespore cells (Azhar et al., 2001). A similar scenario was observed with the cytosolic pH. Cells in M and S phase have a high cytosolic pH and display prestalk tendency and those cells in G2 phase have a low cytosolic pH and display prespore tendency (Aerts et al., 1985, 1987). Therefore, an analysis of cell cycle and the examination of intracellular Ca^2+^ levels and cytosolic pH regulation in diploids could help to improve reconstitution experiments with haploid:diploid populations and pave way for a better, understanding of the evolution of germ-soma like division of labor in *Dictyostelium*.

## Data Availability Statement

The raw data supporting the conclusions of this article will be made available by the authors, without undue reservation.

## Author Contributions

RD and SPS designed the experiments, performed reconstitution experiments, and wrote the manuscript. Both authors contributed to the article and approved the submitted version.

## Conflict of Interest

The authors declare that the research was conducted in the absence of any commercial or financial relationships that could be construed as a potential conflict of interest.

## Abbreviations

AM: aggregation multicellularity
CFSE: carboxy fluorescein succinimydyl ester
DM: division multicellularity
PI: propidium iodide
RFP: red fluorescence protein
UDP: uridine diphosphate.
