# Supplementary material for: Evolution of Reproductive Division of Labor – Lessons Learned From the Social Amoeba Dictyostelium discoideum During Its Multicellular Development
Source: Front Cell Dev Biol. 2021 Mar 4;9:599525. doi: 10.3389/fcell.2021.599525 (PMC7969725; doi:10.3389/fcell.2021.599525)
Supplement: Supplementary file 1 [file Table_1.pdf]

Supplementary Table 1: Ax2 haploid: diploid mixing ratios and methods

Strain Background	Ploidy status		Mixing ratios	
	H	D	H	D
Ax2 wild type	Ax2pTXRFP	Ax2IR150	1	1
			1	9
			2	8
			9	1
			8	2
	Ax2.CFSE stained	Ax2IR150	1	1
			1	9
			2	8
			9	1
			8	2
	Ax2	Ax2IR150. CFSE stained	1	1
			1	9
			2	8
			9	1
			8	2
	Ax2pTXRFP	Ax2IR150. CFSE stained	1	1
			1	9
			2	8
			9	1

			8	2
	Ax2 100% control	Ax2IR150 100% control		
	Ax2pTXRFP 100% control			
	Ax2. CFSE stained 100% control	Ax2IR150. CFSE stained 100% control		

Same pattern was followed for experiments with Ax3 background haploid (Ax3, Ax3pTXRFP, Ax3.CFSE stained) and diploid strains (Ax3IR110 and Ax3IR110.CFSE stained).
